# 4-Acetamido-*N*-(λ^5^-triphenyl­phospho­ranyl­idene)benzene­sulfonamide

**DOI:** 10.1107/S1600536810013620

**Published:** 2010-04-17

**Authors:** Biserka Prugovečki, Marina Marinković, Mladen Vinković, Miljenko Dumić

**Affiliations:** a Laboratory of General and Inorganic Chemistry, Faculty of Science, University of Zagreb, Horvatovac 102a, HR-10000 Zagreb, Croatia; b PLIVA Croatia Ltd, TAPI Research and Development, Prilaz baruna Filipovića 29, HR-10000 Zagreb, Croatia; c Department of Biotechnology, University of Rijeka, S. Krautzeka bb, HR-51000 Rijeka, Croatia

## Abstract

There are two independent mol­ecules per asymmetric unit of the title compound, C_26_H_23_N_2_O_3_PS. Their superposition shows that they differ in the conformation of the CH_3_CO– group and the benzene rings from the triphenyl­phospho­rane group. In the crystal structure, independent mol­ecules are inter­conected by strong N—H⋯O hydrogen bonds, forming infinite chains along the *a* axis.

## Related literature

For related structures, see: Andersen *et al.* (1999 ▶, 2001 ▶, 2004 ▶); Matano *et al.* (2002 ▶); Monkowius *et al.* (2004 ▶); Zhu *et al.* (1997 ▶). For the synthesis, see: Ashley *et al.* (1947 ▶); Khmel’nitzkaya & Mikhel’s (1934 ▶). For structural and synthetic studies of azirine anti­hyperglycaemics, see; Dumić *et al.* (1993 ▶, 1995 ▶); Filić *et al.* (1996 ▶); Orešić *et al.* (2001 ▶); Prugovečki *et al.* (2005 ▶, 2006 ▶); Vinković *et al.* (1993 ▶); Žegarac *et al.* (2010 ▶).
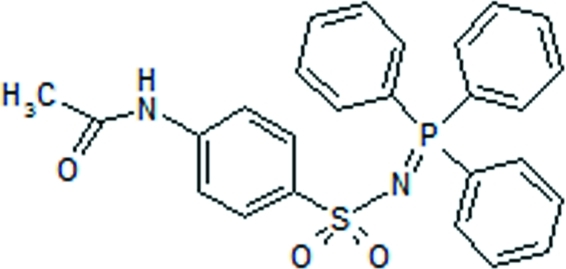


## Experimental

### 

#### Crystal data

C_26_H_23_N_2_O_3_PS
*M* *_r_* = 474.49
Monoclinic, 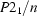

*a* = 15.0419 (10) Å
*b* = 18.6355 (10) Å
*c* = 18.5917 (18) Å
β = 113.413 (10)°
*V* = 4782.4 (6) Å^3^
*Z* = 8
Mo *K*α radiation
μ = 0.23 mm^−1^
*T* = 295 K
0.56 × 0.30 × 0.15 mm

#### Data collection

Oxford Diffraction Xcalibur CCD diffractometer
32329 measured reflections
8441 independent reflections
6309 reflections with *I* > 2σ(*I*)
*R*_int_ = 0.029

#### Refinement

*R*[*F* ^2^ > 2σ(*F* ^2^)] = 0.041
*wR*(*F* ^2^) = 0.106
*S* = 1.04
8441 reflections
595 parameters
H-atom parameters constrained
Δρ_max_ = 0.25 e Å^−3^
Δρ_min_ = −0.28 e Å^−3^

### 

Data collection: *CrysAlis CCD* (Oxford Diffraction, 2003 ▶); cell refinement: *CrysAlis RED* (Oxford Diffraction, 2003 ▶); data reduction: *CrysAlis RED*; program(s) used to solve structure: *SHELXS97* (Sheldrick, 2008 ▶); program(s) used to refine structure: *SHELXL97* (Sheldrick, 2008 ▶); molecular graphics: *PLATON* (Spek, 2009 ▶); software used to prepare material for publication: *SHELXL97*.

## Supplementary Material

Crystal structure: contains datablocks global, I. DOI: 10.1107/S1600536810013620/bg2337sup1.cif

Structure factors: contains datablocks I. DOI: 10.1107/S1600536810013620/bg2337Isup2.hkl

Additional supplementary materials:  crystallographic information; 3D view; checkCIF report

## Figures and Tables

**Table 1:** Hydrogen-bond geometry (Å, °)

*D*—H⋯*A*	*D*—H	H⋯*A*	*D*⋯*A*	*D*—H⋯*A*
N2—H2⋯O2′^i^	0.86	2.11	2.966 (2)	173
N2′—H2′⋯O2^ii^	0.86	2.11	2.961 (2)	174

Symmetry codes: (i) 
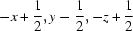
; (ii) 
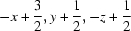
.

**Fig. 1.**
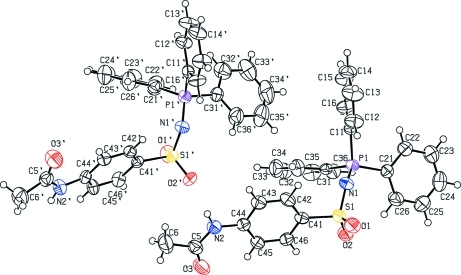
View of the molecule I with the atom labelling scheme. Displacement ellipsoids are drawn at the 50% probability level.

**Fig. 2.**
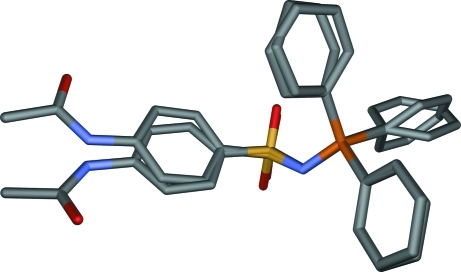
Overlaped structures of independent molecules of compound I, showing structural differences.

**Fig. 3.**
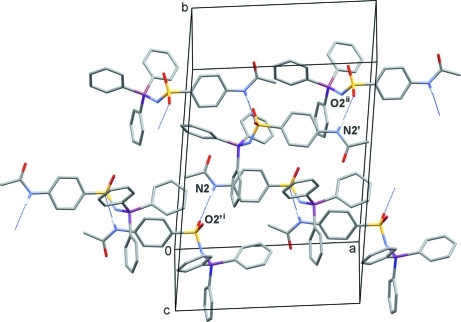
Packing of the molecules in the unit cell. Hydrogen bonds (d(N2—H2···O2'(-x + 1/2, y - 1/2,-z+1/2)=2.966 (2) Å and N2'-H2'···O2(-x + 3/2, y + 1/2,-z+1/2)=2.961 (2) Å) are shown as dotted lines and hydrogen atoms that are not involved in hydrogen bonding are omitted for clarity.

**Fig. 4.**
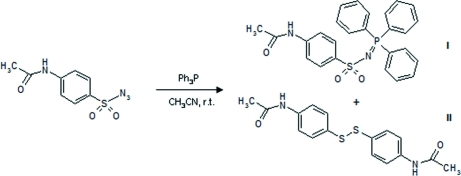
Synthetic route to the molecule I.

## supplementary crystallographic information

### Comment

As a part of our ongoing research on the synthetic and structural studies of 1-sulfonyl-1a,2,6,6a-tetrahydro-1H,4*H*-[1,3]dioxepino[5,6-b]azirine antihyperglycaemics (Dumić *et al.* 1993 ; 1995, Filić *et al.* 1996, Vinković *et al.* 1993, Orešić *et al.* 2001 and Prugovečki *et al.*2005 ; 2006), we required suitable synthons carrying 4-acetylaminobenzenesulfanyl and 4-acetylaminobenzenesulfonyl pattern. Thus, the 4-acetylaminobenzenesulfonyimino-triphenylphosphorane (Title compound, I) and bis(4-acetylaminophenyl) disulfide compound (II) were chosen for this study. We prepared both of them in the same reaction, i.e. by treatment of 4-acetylaminobenzenesulfonylazide with triphenylphosphine in acetonitrile at room temperature (Scheme 1). 4-Acetylaminobenzenesulfonyimino-triphenylphosphorane (Title compound, I) was obtained as colorless prisms (m.p. 495-497 K). Bis(4-acetylaminophenyl) disulfide (compound II) was obtained as a yellow solid (m.p. 485-488 K), i.e. in one of its three known forms; m.ps. 488 K, 454-455 K and 395 K respectively (Khmel'nitzkaya, *et al.* 1934). Its structure and solid state behaviour will be published elsewhere (Žegarac *et al.* 2010).

In the title compound, C_26_H_23_N_2_O_3_PS,(I), there are two independent molecules per asymmetric unit. Their superposition shows that they are different in the conformation of the CH_3_CO group and the benzene rings from the triphenylphosphorane group. In the crystal structure independent molecules are interconected by strong N—H···O hydrogen bonds forming infinite one-dimensional chains along the *a* axis.

### Experimental

Triphenylphosphine (28.18 g, 0.107 mol) was added in small portions to a stirred solution of 4-acetylaminobenzenesulfonyl azide (13.0 g, 50.4 mmol) [prepared according Ashley *et al.* (1947)] in acetonitrile (211 ml) at 273 K. After being stirred for 3 hrs at room temperature, the mixture was concentrated under reduced pressure to dryness. The residue was purified by silicagel column chromatography (dichloromethane-methanol-25 % ammonia, 10:1:0.3 v/v) to afford the title compound (I) as a colorless solid [5.9 g, 22.7 %; m.p. 491-495 K; R_f_ = 0.32 (dichloromethane-methanol- 25 % ammonia, 10:1:0.3 v/v)]. Single crystals suitable for X-ray diffraction were prepared by recrystallization from ethyl acetate-methanol , 1:1 v/v). M.p. 495-497 K. Spectroscopic analysis: IR (KBr) ν_max_/cm^-1^: 3305, 3269, 3190, 3116, 3057, 1691, 1594, 1536, 1485, 1437, 1400, 1372, 1315, 1252, 1194, 1170, 1131, 1085, 1031, 1015, 998, 954, 851, 801, 790, 753, 723, 692, 638, 620. ^1^H NMR (DMSO-d_6_) δ/ppm: 10.12 (s, 1H, NH), 7.52 i 7.40 (d.d., 4H, J=4.3, H-arom.), 7.73-7.68 and 7.59-7.57 (2 m, 15H, H-arom.), 2.06 (s, 3H, CH_3_). ^13^C NMR (DMSO-d_6_) δ/ppm: 141.10 (s), 140.32 (s), 125.93 (d), 117.96 (d) (C arom.), 133.05 (d), 132.60 (d), 128.90 (d), 126.94 (d, J(C—P)=102.9) (C-arom), 24.04 (q, CH_3_).

Evaporation of other selected fractions to dryness afford bis(4-acetylaminophenyl) disulfide (II) as a TLC pure yellow solid [5.7 g, 31.5 % m.p. 485-488 K; R_f_ = 0.26 (dichloromethane-methanol-25 % ammonia, 10:1:0.3 v/v)]. Spectroscopic analysis: IR (KBr) ν_max_/cm^-1^: 3291, 3246, 3178, 3105, 3058, 1681, 1658, 1608, 1593, 1538, 1490, 1397, 1367, 1317, 1292, 1263, 1175, 1121, 1014, 967, 838, 825, 816, 758, 703, 604. ^1^H NMR (DMSO-d_6_) δ/ppm: 10.71 (s, 2H, NH), 7.59 and 7.42 (dd, 8H, H arom, J=8.7), 2.04 (s, 6H, CH_3_). ^13^C NMR (DMSO-d_6_) δ/ppm: 168.50 (s, CO), 139.50 (s), 130.10 (d), 129.10 (s), 119.50 (d) (C arom), 24.20 (q, CH_3_).

### Refinement

H atoms were positioned geometrically, C-H: 0.93-0.96Å, N-H: 0.86Å, and allowed to ride, with U(H)=1.2/1.5× U_eq_(host). In order to avoid beamstop shadowing effects theta(min) was set to 3.24°, with what 20 reflections below this value were left aside the data set.

### Crystal data

**Table:**

C_26_H_23_N_2_O_3_PS	*F*(000) = 1984
*M**_r_* = 474.49	*D*_x_ = 1.318 Mg m^−^^3^
Monoclinic, *P*2_1_/*n*	Mo *K*α radiation, λ = 0.71073 Å
Hall symbol: -P 2yn	Cell parameters from 1548 reflections
*a* = 15.0419 (10) Å	θ = 15–25°
*b* = 18.6355 (10) Å	µ = 0.23 mm^−^^1^
*c* = 18.5917 (18) Å	*T* = 295 K
β = 113.413 (10)°	Plate, colourless
*V* = 4782.4 (6) Å^3^	0.56 × 0.30 × 0.15 mm
*Z* = 8	

### Data collection

**Table:**

Oxford Diffraction Xcalibur CCD diffractometer	6309 reflections with *I* > 2σ(*I*)
Radiation source: fine-focus sealed tube	*R*_int_ = 0.029
graphite	θ_max_ = 25.1°, θ_min_ = 3.2°
CCD scans	*h* = −17→17
32329 measured reflections	*k* = −22→22
8441 independent reflections	*l* = −22→21

### Refinement

**Table:**

Refinement on *F*^2^	Primary atom site location: structure-invariant direct methods
Least-squares matrix: full	Secondary atom site location: difference Fourier map
*R*[*F*^2^ > 2σ(*F*^2^)] = 0.041	Hydrogen site location: inferred from neighbouring sites
*wR*(*F*^2^) = 0.106	H-atom parameters constrained
*S* = 1.04	*w* = 1/[σ^2^(*F*_o_^2^) + (0.0611*P*)^2^ + 0.0767*P*] where *P* = (*F*_o_^2^ + 2*F*_c_^2^)/3
8441 reflections	(Δ/σ)_max_ = 0.001
595 parameters	Δρ_max_ = 0.25 e Å^−^^3^
0 restraints	Δρ_min_ = −0.28 e Å^−^^3^

### Fractional atomic coordinates and isotropic or equivalent isotropic displacement parameters (Å^2^)

**Table:**

	*x*	*y*	*z*	*U*_iso_*/*U*_eq_	
S1	0.60435 (4)	0.26549 (3)	0.06832 (3)	0.03823 (15)	
P1	0.73384 (4)	0.22036 (3)	0.22228 (3)	0.03282 (14)	
O1	0.63908 (11)	0.33781 (8)	0.08560 (10)	0.0540 (4)	
O2	0.59940 (12)	0.23771 (9)	−0.00544 (8)	0.0545 (4)	
O3	0.15446 (12)	0.38553 (10)	0.01877 (11)	0.0640 (5)	
N1	0.66148 (12)	0.20983 (9)	0.13335 (10)	0.0381 (4)	
N2	0.19539 (13)	0.26777 (10)	0.03546 (10)	0.0439 (5)	
H2	0.1725	0.2259	0.0379	0.053*	
C5	0.13239 (17)	0.32332 (14)	0.02196 (13)	0.0492 (6)	
C6	0.0322 (2)	0.30213 (17)	0.0123 (2)	0.0857 (10)	
H6A	0.0295	0.2985	0.0629	0.128*	
H6B	0.0159	0.2566	−0.0139	0.128*	
H6C	−0.0130	0.3378	−0.0183	0.128*	
C11	0.74745 (15)	0.13304 (11)	0.26552 (12)	0.0364 (5)	
C12	0.72650 (19)	0.07279 (12)	0.21875 (15)	0.0559 (7)	
H12	0.7056	0.0773	0.1647	0.067*	
C13	0.7369 (2)	0.00600 (13)	0.25234 (19)	0.0715 (8)	
H13	0.7216	−0.0346	0.2207	0.086*	
C14	0.76928 (19)	−0.00145 (13)	0.33235 (17)	0.0595 (7)	
H14	0.7773	−0.0469	0.3546	0.071*	
C15	0.78979 (19)	0.05756 (13)	0.37871 (15)	0.0576 (7)	
H15	0.8114	0.0527	0.4328	0.069*	
C16	0.77845 (18)	0.12463 (12)	0.34556 (13)	0.0492 (6)	
H16	0.7919	0.1650	0.3775	0.059*	
C21	0.85231 (15)	0.25325 (11)	0.23595 (12)	0.0372 (5)	
C22	0.93602 (18)	0.22040 (15)	0.28608 (15)	0.0589 (7)	
H22	0.9322	0.1797	0.3136	0.071*	
C23	1.02521 (19)	0.24721 (17)	0.29590 (17)	0.0716 (8)	
H23	1.0811	0.2241	0.3295	0.086*	
C24	1.03252 (19)	0.30698 (16)	0.25716 (17)	0.0641 (7)	
H24	1.0930	0.3255	0.2647	0.077*	
C25	0.9506 (2)	0.33945 (15)	0.20712 (18)	0.0690 (8)	
H25	0.9553	0.3802	0.1800	0.083*	
C26	0.86062 (18)	0.31328 (13)	0.19594 (16)	0.0562 (7)	
H26	0.8052	0.3361	0.1611	0.067*	
C31	0.68889 (15)	0.27629 (11)	0.27907 (12)	0.0352 (5)	
C32	0.59488 (17)	0.26534 (13)	0.27220 (14)	0.0498 (6)	
H32	0.5557	0.2321	0.2363	0.060*	
C33	0.5591 (2)	0.30353 (15)	0.31856 (16)	0.0621 (7)	
H33	0.4961	0.2957	0.3142	0.074*	
C34	0.6167 (2)	0.35311 (14)	0.37095 (15)	0.0592 (7)	
H34	0.5927	0.3789	0.4021	0.071*	
C35	0.7090 (2)	0.36480 (13)	0.37737 (14)	0.0578 (7)	
H35	0.7473	0.3988	0.4127	0.069*	
C36	0.74610 (18)	0.32633 (12)	0.33204 (13)	0.0462 (6)	
H36	0.8094	0.3342	0.3371	0.055*	
C41	0.48472 (15)	0.26548 (11)	0.06320 (11)	0.0345 (5)	
C42	0.43550 (17)	0.20264 (11)	0.06204 (13)	0.0442 (6)	
H42	0.4667	0.1587	0.0668	0.053*	
C43	0.34066 (17)	0.20470 (11)	0.05396 (13)	0.0435 (5)	
H43	0.3082	0.1622	0.0538	0.052*	
C44	0.29302 (15)	0.26972 (11)	0.04594 (11)	0.0368 (5)	
C45	0.34292 (16)	0.33258 (12)	0.04923 (13)	0.0438 (5)	
H45	0.3123	0.3766	0.0457	0.053*	
C46	0.43846 (16)	0.32999 (11)	0.05761 (13)	0.0419 (5)	
H46	0.4718	0.3725	0.0596	0.050*	
S1'	0.39565 (4)	0.60029 (3)	0.37549 (3)	0.03796 (15)	
P1'	0.29976 (4)	0.48985 (3)	0.27043 (3)	0.03572 (15)	
O1'	0.36387 (11)	0.64762 (8)	0.30907 (9)	0.0540 (4)	
O2'	0.37677 (11)	0.62497 (8)	0.44208 (9)	0.0499 (4)	
O3'	0.86803 (13)	0.47540 (10)	0.53859 (12)	0.0703 (5)	
N1'	0.35642 (13)	0.52178 (9)	0.35569 (10)	0.0399 (4)	
N2'	0.82514 (13)	0.59181 (10)	0.50987 (11)	0.0456 (5)	
H2'	0.8495	0.6340	0.5122	0.055*	
C03'	0.0174 (2)	0.52015 (17)	0.12570 (19)	0.0807 (10)	
H23'	−0.0241	0.5092	0.0745	0.097*	
C5'	0.89003 (17)	0.53794 (15)	0.54146 (13)	0.0493 (6)	
C6'	0.99288 (18)	0.56331 (16)	0.58155 (16)	0.0698 (8)	
H6'1	1.0212	0.5429	0.6332	0.105*	
H6'2	0.9940	0.6147	0.5853	0.105*	
H6'3	1.0292	0.5484	0.5518	0.105*	
C11'	0.30394 (14)	0.39466 (11)	0.28443 (12)	0.0348 (5)	
C12'	0.28480 (17)	0.34757 (12)	0.22329 (13)	0.0475 (6)	
H12'	0.2732	0.3650	0.1735	0.057*	
C13'	0.28260 (19)	0.27466 (13)	0.23523 (14)	0.0560 (7)	
H13'	0.2698	0.2431	0.1936	0.067*	
C14'	0.29939 (18)	0.24874 (13)	0.30821 (14)	0.0531 (6)	
H14'	0.2978	0.1996	0.3161	0.064*	
C15'	0.31851 (19)	0.29467 (13)	0.36925 (14)	0.0554 (7)	
H15'	0.3294	0.2767	0.4187	0.066*	
C16'	0.32181 (18)	0.36738 (12)	0.35837 (13)	0.0492 (6)	
H16'	0.3360	0.3984	0.4006	0.059*	
C21'	0.17467 (16)	0.51602 (12)	0.23034 (13)	0.0429 (5)	
C22'	0.11238 (18)	0.49844 (14)	0.15399 (16)	0.0615 (7)	
H22'	0.1352	0.4722	0.1223	0.074*	
C24'	−0.0166 (2)	0.55839 (19)	0.1731 (2)	0.0878 (10)	
H24'	−0.0808	0.5734	0.1535	0.105*	
C25'	0.0431 (2)	0.57394 (18)	0.2480 (2)	0.0856 (10)	
H25'	0.0192	0.5986	0.2800	0.103*	
C26'	0.13908 (18)	0.55348 (14)	0.27692 (16)	0.0609 (7)	
H26'	0.1800	0.5651	0.3281	0.073*	
C31'	0.35067 (17)	0.51011 (12)	0.19990 (13)	0.0437 (5)	
C32'	0.43991 (19)	0.48131 (15)	0.21220 (17)	0.0645 (7)	
H32'	0.4698	0.4499	0.2538	0.077*	
C33'	0.4851 (2)	0.4989 (2)	0.1627 (2)	0.0864 (10)	
H33'	0.5454	0.4794	0.1714	0.104*	
C34'	0.4426 (3)	0.5441 (2)	0.1020 (2)	0.0937 (12)	
H34'	0.4735	0.5553	0.0689	0.112*	
C35'	0.3549 (3)	0.57337 (19)	0.08893 (19)	0.0904 (11)	
H35'	0.3259	0.6045	0.0470	0.108*	
C36'	0.3084 (2)	0.55685 (15)	0.13822 (16)	0.0690 (8)	
H36'	0.2486	0.5773	0.1295	0.083*	
C41'	0.52275 (15)	0.59244 (11)	0.40911 (12)	0.0336 (5)	
C42'	0.57060 (15)	0.53071 (11)	0.44354 (12)	0.0355 (5)	
H42'	0.5352	0.4904	0.4455	0.043*	
C43'	0.67037 (16)	0.52770 (11)	0.47535 (12)	0.0374 (5)	
H43'	0.7019	0.4853	0.4977	0.045*	
C44'	0.72346 (15)	0.58761 (11)	0.47384 (12)	0.0361 (5)	
C45'	0.67459 (16)	0.64967 (12)	0.43796 (13)	0.0430 (5)	
H45'	0.7098	0.6900	0.4358	0.052*	
C46'	0.57519 (16)	0.65226 (12)	0.40549 (12)	0.0408 (5)	
H46'	0.5433	0.6940	0.3813	0.049*	

### Atomic displacement parameters (Å^2^)

**Table:**

	*U*^11^	*U*^22^	*U*^33^	*U*^12^	*U*^13^	*U*^23^
S1	0.0389 (3)	0.0373 (3)	0.0383 (3)	0.0041 (2)	0.0152 (2)	0.0027 (2)
P1	0.0348 (3)	0.0288 (3)	0.0341 (3)	0.0015 (2)	0.0129 (2)	−0.0013 (2)
O1	0.0462 (10)	0.0352 (9)	0.0782 (12)	−0.0018 (7)	0.0221 (8)	0.0049 (8)
O2	0.0603 (11)	0.0717 (11)	0.0345 (8)	0.0177 (9)	0.0221 (8)	0.0050 (8)
O3	0.0454 (11)	0.0511 (11)	0.0861 (13)	0.0109 (9)	0.0160 (9)	0.0114 (10)
N1	0.0396 (11)	0.0365 (10)	0.0349 (10)	0.0054 (8)	0.0113 (8)	−0.0015 (8)
N2	0.0400 (11)	0.0431 (11)	0.0482 (11)	−0.0026 (9)	0.0170 (9)	0.0012 (9)
C5	0.0395 (14)	0.0572 (16)	0.0450 (14)	0.0049 (13)	0.0104 (11)	0.0061 (12)
C6	0.0487 (18)	0.086 (2)	0.122 (3)	0.0049 (16)	0.0332 (18)	0.009 (2)
C11	0.0347 (12)	0.0310 (11)	0.0430 (12)	0.0026 (9)	0.0149 (10)	−0.0007 (10)
C12	0.0745 (19)	0.0366 (13)	0.0501 (15)	0.0027 (13)	0.0178 (13)	−0.0059 (11)
C13	0.090 (2)	0.0291 (14)	0.085 (2)	0.0007 (14)	0.0236 (18)	−0.0074 (14)
C14	0.0590 (17)	0.0377 (14)	0.077 (2)	0.0026 (12)	0.0215 (15)	0.0157 (13)
C15	0.0685 (18)	0.0488 (16)	0.0534 (15)	0.0060 (13)	0.0220 (13)	0.0142 (13)
C16	0.0641 (17)	0.0369 (13)	0.0426 (13)	0.0002 (12)	0.0169 (12)	0.0032 (11)
C21	0.0369 (13)	0.0368 (12)	0.0389 (12)	0.0020 (10)	0.0162 (10)	−0.0053 (10)
C22	0.0427 (15)	0.0688 (18)	0.0598 (16)	−0.0004 (13)	0.0146 (13)	0.0184 (14)
C23	0.0377 (16)	0.095 (2)	0.0728 (19)	0.0017 (15)	0.0120 (14)	0.0172 (17)
C24	0.0409 (16)	0.080 (2)	0.0736 (19)	−0.0127 (14)	0.0248 (14)	−0.0107 (16)
C25	0.0576 (19)	0.0526 (16)	0.100 (2)	−0.0094 (14)	0.0350 (17)	0.0108 (16)
C26	0.0421 (15)	0.0423 (14)	0.0804 (18)	0.0015 (11)	0.0203 (13)	0.0119 (13)
C31	0.0427 (13)	0.0300 (11)	0.0331 (11)	0.0034 (10)	0.0151 (10)	0.0008 (9)
C32	0.0465 (15)	0.0543 (15)	0.0506 (14)	−0.0021 (12)	0.0214 (12)	−0.0123 (12)
C33	0.0537 (17)	0.0761 (19)	0.0657 (17)	0.0099 (14)	0.0336 (14)	−0.0046 (15)
C34	0.084 (2)	0.0533 (16)	0.0495 (15)	0.0160 (15)	0.0357 (15)	−0.0042 (12)
C35	0.082 (2)	0.0431 (14)	0.0485 (15)	−0.0036 (13)	0.0263 (14)	−0.0140 (12)
C36	0.0547 (15)	0.0374 (13)	0.0479 (14)	−0.0033 (11)	0.0218 (12)	−0.0064 (11)
C41	0.0376 (12)	0.0326 (12)	0.0310 (11)	0.0018 (10)	0.0110 (9)	−0.0007 (9)
C42	0.0477 (15)	0.0297 (12)	0.0518 (14)	0.0052 (10)	0.0162 (11)	−0.0008 (10)
C43	0.0452 (14)	0.0321 (12)	0.0518 (14)	−0.0049 (10)	0.0176 (11)	0.0000 (10)
C44	0.0356 (12)	0.0425 (13)	0.0293 (11)	0.0003 (10)	0.0097 (9)	0.0001 (10)
C45	0.0480 (14)	0.0321 (12)	0.0547 (14)	0.0063 (11)	0.0240 (12)	0.0057 (10)
C46	0.0449 (14)	0.0313 (12)	0.0513 (14)	−0.0017 (10)	0.0210 (11)	0.0020 (10)
S1'	0.0363 (3)	0.0317 (3)	0.0452 (3)	0.0027 (2)	0.0155 (2)	−0.0002 (2)
P1'	0.0336 (3)	0.0361 (3)	0.0358 (3)	0.0005 (2)	0.0120 (2)	0.0008 (2)
O1'	0.0474 (10)	0.0444 (10)	0.0613 (11)	0.0077 (8)	0.0123 (8)	0.0164 (8)
O2'	0.0499 (10)	0.0455 (9)	0.0630 (10)	−0.0013 (8)	0.0318 (8)	−0.0155 (8)
O3'	0.0495 (11)	0.0474 (11)	0.1054 (16)	0.0086 (9)	0.0216 (10)	0.0047 (10)
N1'	0.0435 (11)	0.0343 (10)	0.0388 (10)	−0.0022 (8)	0.0131 (8)	−0.0006 (8)
N2'	0.0366 (11)	0.0444 (11)	0.0549 (12)	−0.0016 (9)	0.0174 (9)	0.0056 (9)
C03'	0.0468 (18)	0.084 (2)	0.080 (2)	0.0057 (16)	−0.0078 (16)	0.0192 (18)
C5'	0.0418 (15)	0.0585 (17)	0.0466 (14)	0.0058 (13)	0.0166 (11)	−0.0009 (12)
C6'	0.0415 (16)	0.080 (2)	0.078 (2)	0.0046 (15)	0.0131 (14)	−0.0015 (16)
C11'	0.0310 (11)	0.0374 (12)	0.0349 (11)	−0.0026 (9)	0.0119 (9)	−0.0030 (9)
C12'	0.0583 (16)	0.0457 (14)	0.0362 (12)	−0.0105 (12)	0.0164 (11)	−0.0010 (10)
C13'	0.0768 (19)	0.0414 (14)	0.0445 (14)	−0.0163 (13)	0.0185 (13)	−0.0119 (11)
C14'	0.0650 (17)	0.0378 (13)	0.0519 (15)	−0.0100 (12)	0.0183 (13)	−0.0007 (12)
C15'	0.0785 (19)	0.0460 (15)	0.0393 (13)	−0.0033 (13)	0.0210 (13)	0.0074 (11)
C16'	0.0684 (17)	0.0405 (13)	0.0370 (13)	−0.0005 (12)	0.0192 (12)	−0.0059 (10)
C21'	0.0363 (13)	0.0423 (13)	0.0479 (14)	0.0040 (10)	0.0145 (11)	0.0066 (11)
C22'	0.0469 (16)	0.0644 (18)	0.0604 (17)	0.0041 (13)	0.0078 (13)	−0.0002 (13)
C24'	0.0404 (17)	0.095 (3)	0.120 (3)	0.0178 (17)	0.0229 (19)	0.020 (2)
C25'	0.058 (2)	0.102 (3)	0.105 (3)	0.0288 (19)	0.041 (2)	0.011 (2)
C26'	0.0495 (16)	0.0701 (18)	0.0644 (17)	0.0122 (14)	0.0240 (13)	0.0032 (14)
C31'	0.0496 (14)	0.0414 (13)	0.0436 (13)	−0.0076 (11)	0.0224 (11)	−0.0033 (10)
C32'	0.0516 (17)	0.0740 (19)	0.0761 (19)	−0.0051 (14)	0.0341 (15)	−0.0010 (15)
C33'	0.070 (2)	0.107 (3)	0.106 (3)	−0.021 (2)	0.061 (2)	−0.021 (2)
C34'	0.124 (3)	0.101 (3)	0.090 (3)	−0.052 (3)	0.078 (3)	−0.025 (2)
C35'	0.131 (3)	0.089 (2)	0.068 (2)	−0.014 (2)	0.058 (2)	0.0175 (18)
C36'	0.087 (2)	0.0662 (18)	0.0634 (18)	0.0045 (16)	0.0396 (16)	0.0163 (15)
C41'	0.0374 (12)	0.0333 (11)	0.0335 (11)	0.0008 (9)	0.0178 (9)	−0.0021 (9)
C42'	0.0404 (13)	0.0269 (11)	0.0416 (12)	−0.0018 (9)	0.0190 (10)	−0.0027 (9)
C43'	0.0420 (13)	0.0318 (12)	0.0410 (12)	0.0058 (10)	0.0193 (10)	0.0011 (9)
C44'	0.0359 (12)	0.0413 (13)	0.0348 (11)	0.0009 (10)	0.0178 (10)	0.0019 (9)
C45'	0.0401 (13)	0.0415 (13)	0.0527 (14)	−0.0026 (11)	0.0239 (11)	0.0093 (11)
C46'	0.0451 (14)	0.0353 (12)	0.0450 (13)	0.0052 (10)	0.0210 (11)	0.0106 (10)

### Geometric parameters (Å, °)

**Table:**

S1—O1	1.4348 (16)	S1'—O1'	1.4361 (15)
S1—O2	1.4398 (15)	S1'—O2'	1.4506 (15)
S1—N1	1.5655 (17)	S1'—N1'	1.5658 (18)
S1—C41	1.764 (2)	S1'—C41'	1.766 (2)
P1—N1	1.5896 (18)	P1'—N1'	1.5872 (18)
P1—C11	1.790 (2)	P1'—C11'	1.790 (2)
P1—C31	1.796 (2)	P1'—C21'	1.794 (2)
P1—C21	1.804 (2)	P1'—C31'	1.802 (2)
O3—C5	1.214 (3)	O3'—C5'	1.207 (3)
N2—C5	1.358 (3)	N2'—C5'	1.360 (3)
N2—C44	1.403 (3)	N2'—C44'	1.407 (3)
N2—H2	0.8605	N2'—H2'	0.8606
C5—C6	1.498 (3)	C03'—C22'	1.373 (4)
C6—H6A	0.9600	C03'—C24'	1.379 (5)
C6—H6B	0.9600	C03'—H23'	0.9300
C6—H6C	0.9600	C5'—C6'	1.502 (3)
C11—C16	1.380 (3)	C6'—H6'1	0.9600
C11—C12	1.378 (3)	C6'—H6'2	0.9600
C12—C13	1.373 (3)	C6'—H6'3	0.9600
C12—H12	0.9300	C11'—C12'	1.373 (3)
C13—C14	1.376 (4)	C11'—C16'	1.388 (3)
C13—H13	0.9300	C12'—C13'	1.379 (3)
C14—C15	1.355 (3)	C12'—H12'	0.9300
C14—H14	0.9300	C13'—C14'	1.366 (3)
C15—C16	1.374 (3)	C13'—H13'	0.9300
C15—H15	0.9300	C14'—C15'	1.358 (3)
C16—H16	0.9300	C14'—H14'	0.9300
C21—C22	1.377 (3)	C15'—C16'	1.374 (3)
C21—C26	1.376 (3)	C15'—H15'	0.9300
C22—C23	1.374 (3)	C16'—H16'	0.9300
C22—H22	0.9300	C21'—C26'	1.376 (3)
C23—C24	1.354 (4)	C21'—C22'	1.394 (3)
C23—H23	0.9300	C22'—H22'	0.9300
C24—C25	1.357 (4)	C24'—C25'	1.355 (5)
C24—H24	0.9300	C24'—H24'	0.9300
C25—C26	1.374 (3)	C25'—C26'	1.379 (4)
C25—H25	0.9300	C25'—H25'	0.9300
C26—H26	0.9300	C26'—H26'	0.9300
C31—C32	1.384 (3)	C31'—C32'	1.378 (3)
C31—C36	1.381 (3)	C31'—C36'	1.378 (3)
C32—C33	1.381 (3)	C32'—C33'	1.383 (4)
C32—H32	0.9300	C32'—H32'	0.9300
C33—C34	1.372 (4)	C33'—C34'	1.349 (5)
C33—H33	0.9300	C33'—H33'	0.9300
C34—C35	1.362 (4)	C34'—C35'	1.357 (5)
C34—H34	0.9300	C34'—H34'	0.9300
C35—C36	1.382 (3)	C35'—C36'	1.390 (4)
C35—H35	0.9300	C35'—H35'	0.9300
C36—H36	0.9300	C36'—H36'	0.9300
C41—C46	1.372 (3)	C41'—C42'	1.373 (3)
C41—C42	1.381 (3)	C41'—C46'	1.383 (3)
C42—C43	1.374 (3)	C42'—C43'	1.378 (3)
C42—H42	0.9300	C42'—H42'	0.9300
C43—C44	1.385 (3)	C43'—C44'	1.379 (3)
C43—H43	0.9300	C43'—H43'	0.9300
C44—C45	1.379 (3)	C44'—C45'	1.390 (3)
C45—C46	1.383 (3)	C45'—C46'	1.373 (3)
C45—H45	0.9300	C45'—H45'	0.9300
C46—H46	0.9300	C46'—H46'	0.9300
			
O1—S1—O2	115.31 (10)	O1'—S1'—O2'	115.34 (10)
O1—S1—N1	114.21 (10)	O1'—S1'—N1'	113.96 (10)
O2—S1—N1	107.58 (9)	O2'—S1'—N1'	108.28 (9)
O1—S1—C41	106.56 (10)	O1'—S1'—C41'	107.39 (10)
O2—S1—C41	106.44 (10)	O2'—S1'—C41'	106.02 (9)
N1—S1—C41	106.06 (10)	N1'—S1'—C41'	105.05 (10)
N1—P1—C11	105.29 (9)	N1'—P1'—C11'	104.62 (9)
N1—P1—C31	114.90 (9)	N1'—P1'—C21'	111.80 (10)
C11—P1—C31	105.43 (9)	C11'—P1'—C21'	107.47 (10)
N1—P1—C21	114.72 (9)	N1'—P1'—C31'	115.68 (10)
C11—P1—C21	108.37 (10)	C11'—P1'—C31'	108.23 (10)
C31—P1—C21	107.54 (10)	C21'—P1'—C31'	108.63 (11)
S1—N1—P1	131.33 (11)	S1'—N1'—P1'	126.00 (11)
C5—N2—C44	128.4 (2)	C5'—N2'—C44'	128.5 (2)
C5—N2—H2	115.8	C5'—N2'—H2'	115.7
C44—N2—H2	115.8	C44'—N2'—H2'	115.8
O3—C5—N2	123.6 (2)	C22'—C03'—C24'	120.1 (3)
O3—C5—C6	121.8 (2)	C22'—C03'—H23'	120.0
N2—C5—C6	114.6 (2)	C24'—C03'—H23'	119.9
C5—C6—H6A	109.5	O3'—C5'—N2'	123.8 (2)
C5—C6—H6B	109.6	O3'—C5'—C6'	122.4 (2)
H6A—C6—H6B	109.5	N2'—C5'—C6'	113.8 (2)
C5—C6—H6C	109.3	C5'—C6'—H6'1	109.4
H6A—C6—H6C	109.5	C5'—C6'—H6'2	109.6
H6B—C6—H6C	109.5	H6'1—C6'—H6'2	109.5
C16—C11—C12	118.8 (2)	C5'—C6'—H6'3	109.5
C16—C11—P1	121.12 (17)	H6'1—C6'—H6'3	109.5
C12—C11—P1	120.04 (17)	H6'2—C6'—H6'3	109.5
C13—C12—C11	119.7 (2)	C12'—C11'—C16'	118.7 (2)
C13—C12—H12	120.1	C12'—C11'—P1'	121.96 (16)
C11—C12—H12	120.2	C16'—C11'—P1'	119.25 (16)
C12—C13—C14	120.7 (3)	C11'—C12'—C13'	120.5 (2)
C12—C13—H13	119.7	C11'—C12'—H12'	119.7
C14—C13—H13	119.6	C13'—C12'—H12'	119.8
C15—C14—C13	119.9 (2)	C14'—C13'—C12'	120.1 (2)
C15—C14—H14	120.0	C14'—C13'—H13'	119.9
C13—C14—H14	120.0	C12'—C13'—H13'	120.0
C14—C15—C16	119.8 (2)	C15'—C14'—C13'	120.1 (2)
C14—C15—H15	120.2	C15'—C14'—H14'	120.0
C16—C15—H15	120.0	C13'—C14'—H14'	119.9
C15—C16—C11	121.0 (2)	C14'—C15'—C16'	120.4 (2)
C15—C16—H16	119.5	C14'—C15'—H15'	119.7
C11—C16—H16	119.4	C16'—C15'—H15'	119.9
C22—C21—C26	118.2 (2)	C15'—C16'—C11'	120.2 (2)
C22—C21—P1	122.05 (18)	C15'—C16'—H16'	119.9
C26—C21—P1	119.78 (17)	C11'—C16'—H16'	119.9
C23—C22—C21	120.7 (2)	C26'—C21'—C22'	119.2 (2)
C23—C22—H22	119.6	C26'—C21'—P1'	118.85 (18)
C21—C22—H22	119.7	C22'—C21'—P1'	121.92 (19)
C24—C23—C22	120.6 (3)	C03'—C22'—C21'	119.8 (3)
C24—C23—H23	119.7	C03'—C22'—H22'	120.1
C22—C23—H23	119.7	C21'—C22'—H22'	120.1
C23—C24—C25	119.2 (3)	C25'—C24'—C03'	120.3 (3)
C23—C24—H24	120.4	C25'—C24'—H24'	119.8
C25—C24—H24	120.4	C03'—C24'—H24'	119.9
C24—C25—C26	121.2 (3)	C24'—C25'—C26'	120.3 (3)
C24—C25—H25	119.4	C24'—C25'—H25'	119.8
C26—C25—H25	119.4	C26'—C25'—H25'	119.8
C25—C26—C21	120.1 (2)	C21'—C26'—C25'	120.2 (3)
C25—C26—H26	119.9	C21'—C26'—H26'	119.8
C21—C26—H26	119.9	C25'—C26'—H26'	119.9
C32—C31—C36	119.3 (2)	C32'—C31'—C36'	118.8 (2)
C32—C31—P1	118.03 (16)	C32'—C31'—P1'	117.74 (19)
C36—C31—P1	122.53 (17)	C36'—C31'—P1'	123.3 (2)
C33—C32—C31	120.2 (2)	C31'—C32'—C33'	120.1 (3)
C33—C32—H32	119.9	C31'—C32'—H32'	120.0
C31—C32—H32	119.8	C33'—C32'—H32'	119.9
C34—C33—C32	119.8 (2)	C34'—C33'—C32'	120.7 (3)
C34—C33—H33	120.1	C34'—C33'—H33'	119.6
C32—C33—H33	120.1	C32'—C33'—H33'	119.7
C35—C34—C33	120.3 (2)	C33'—C34'—C35'	120.3 (3)
C35—C34—H34	119.9	C33'—C34'—H34'	119.9
C33—C34—H34	119.9	C35'—C34'—H34'	119.8
C34—C35—C36	120.5 (2)	C34'—C35'—C36'	120.1 (3)
C34—C35—H35	119.7	C34'—C35'—H35'	119.9
C36—C35—H35	119.8	C36'—C35'—H35'	120.0
C31—C36—C35	119.8 (2)	C31'—C36'—C35'	120.1 (3)
C31—C36—H36	120.1	C31'—C36'—H36'	120.0
C35—C36—H36	120.1	C35'—C36'—H36'	119.9
C46—C41—C42	119.3 (2)	C42'—C41'—C46'	119.7 (2)
C46—C41—S1	118.67 (16)	C42'—C41'—S1'	121.85 (16)
C42—C41—S1	122.01 (16)	C46'—C41'—S1'	118.32 (16)
C43—C42—C41	120.3 (2)	C41'—C42'—C43'	120.91 (19)
C43—C42—H42	119.8	C41'—C42'—H42'	119.5
C41—C42—H42	119.8	C43'—C42'—H42'	119.5
C42—C43—C44	120.4 (2)	C44'—C43'—C42'	119.9 (2)
C42—C43—H43	119.8	C44'—C43'—H43'	120.0
C44—C43—H43	119.8	C42'—C43'—H43'	120.1
C45—C44—C43	119.3 (2)	C43'—C44'—C45'	118.9 (2)
C45—C44—N2	123.3 (2)	C43'—C44'—N2'	124.32 (19)
C43—C44—N2	117.41 (19)	C45'—C44'—N2'	116.72 (19)
C44—C45—C46	119.9 (2)	C46'—C45'—C44'	121.1 (2)
C44—C45—H45	120.0	C46'—C45'—H45'	119.5
C46—C45—H45	120.1	C44'—C45'—H45'	119.4
C41—C46—C45	120.8 (2)	C45'—C46'—C41'	119.5 (2)
C41—C46—H46	119.6	C45'—C46'—H46'	120.3
C45—C46—H46	119.6	C41'—C46'—H46'	120.2

### Hydrogen-bond geometry (Å, °)

**Table:**

*D*—H···*A*	*D*—H	H···*A*	*D*···*A*	*D*—H···*A*
N2—H2···O2'^i^	0.86	2.11	2.966 (2)	173
N2'—H2'···O2^ii^	0.86	2.11	2.961 (2)	174

Symmetry codes: (i) −*x*+1/2, *y*−1/2, −*z*+1/2; (ii) −*x*+3/2, *y*+1/2, −*z*+1/2.
